# On nonparametric estimating ROC curve based on non-uniform rational B-spline

**DOI:** 10.1371/journal.pone.0330175

**Published:** 2025-08-20

**Authors:** Mahmut Sami Erdoğan

**Affiliations:** Department of Statistics, Faculty of Engineering and Natural Sciences, Istanbul Medeniyet University, Istanbul, Turkey; The University of British Columbia, CANADA

## Abstract

The receiver operating characteristic (ROC) curve is a commonly used statistical method to assess the efficacy of a diagnostic test or biomarker measured on a continuous scale. This work presents a versatile approach using a non-uniform rational B-spline (NURBS) for estimating the ROC curve. This approach uses control points, weights, and the knot sequence to more accurately estimate the true ROC curve. The new method applies linear constraints to the NURBS basis function coefficients to smooth the empirical ROC curve and guarantee a non-decreasing function. Moreover, as a specific case, a NURBS curve devoid of interior knots simplifies to the Bernstein polynomial when all weight values are equal. We conduct Monte Carlo simulation studies to evaluate how well the NURBS-based estimator works in different scenarios. We compare our estimator to the empirical ROC, the kernel-based ROC, and Bernstein polynomial estimators in terms of the averaged squared errors. We also apply our method to two real medical datasets, such as metastatic kidney cancer and diffuse large B-cell lymphoma datasets. According to the findings from both the real and simulated data, the NURBS method is a powerful alternative for estimating the ROC curve.

## Introduction

In biostatistical and epidemiological studies, it is critical to evaluate how well medical biomarkers and clinical diagnosis tests work. Typically, researchers use the receiver operating characteristic (ROC) curve to examine the classification accuracy of biomarkers or diagnostic tests. The ROC curve is a widely utilized statistical method to evaluate the ability of biomarkers and diagnostic tests to distinguish when the results are continuous. This two-dimensional curve visualizes the relationship between true positives (sensitivity) and false positives (1-specificity) at different threshold points for the biomarker. This visualization assists in determining the ideal threshold for clinical use. The ROC curve is widely used in numerous fields, such as radiography and medical imaging, biopharmaceutical research and drug effectiveness, epidemiology, and systems for making clinical decisions. The estimation of the ROC curve has been studied extensively in the literature. For a comprehensive review of the most recent developments, we refer to [1–6].

It is generally assumed that a higher biomarker value indicates a higher probability or severity of the disease, so we can label someone as having the disease if their biomarker is above a certain level *s* and not having it if it’s below that. More specifically, we suppose that $X_{1},X_{2},\ldots ,X_{n_{1}}$ and $Y_{1},Y_{2},\ldots ,Y_{n_{2}}$ respectively represent two independent random samples associated with diagnostic test outcomes for a healthy population and a diseased population, where *X* and *Y* denote the corresponding random variables for healthy and diseased individuals. Here, *D* denotes an individual’s status: *D* = 1 denotes disease presence, and *D* = 0 indicates health. Based on this idea, the sensitivity is defined as Se(s)=1−G(s), and the specificity is defined as Sp(s)=F(s), where *F* and *G* represent the cumulative distribution functions (CDFs) of *X* and *Y*, respectively. Next, the ROC curve is defined as a graph of *Se*(*s*) against 1–*Sp*(*s*) for −∞≤s≤∞, and it can be expressed as

$$ROC(t)=1-G(F^{-1}(1-t)),t\in [0,1].$$ (1)

Researchers have developed many estimators for the ROC curve from a parametric, non-parametric, semi-parametric, and Bayesian statistical perspective (see [7] for a detailed review). The non-parametric ROC estimation methods do not necessitate any assumptions on the distributions of diagnostic test results and enhance flexibility in data modeling. The empirical estimator is currently the most utilized non-parametric technique. The empirical estimator of the ROC curve is given by

$$ROC_{n}(t)=1-G_{n_{2}}(F_{n_{1}}^{-1}(1-t)),t\in [0,1]$$ (2)

where $n=n_{1}\;+\;n_{2}$ and $F_{n_{1}}$ and $G_{n_{2}}$ are the empirical CDFs of the samples $\{X_{i}{\}}_{i=1}^{n_{1}}$ and $\{Y_{j}{\}}_{j=1}^{n_{2}}$, respectively. [8] demonstrated that the empirical ROC curve has many similarities with the empirical distribution function and uniformly converges to the true ROC curve as the sample size increases. However, a primary drawback is the inability to guarantee curve smoothness due to its step function characteristic. The ROC curve’s estimated shape can appear jagged, especially when there are small sample sizes, which can lead to significant differences in how sensitivity and specificity are understood in a clinical setting, even with similar threshold values. Kernel-based smoothing techniques have been proposed to address this smoothness problem. Kernel-based ROC estimators smooth the ROC curve, and research shows that the smoothed curve performs better than an unsmooth one (see [9–12]). However, kernel methods tend to have higher error rates, especially at the boundaries, and finding the best bandwidth is difficult, making their application in clinical settings challenging [13,14].

Recent research indicates that ROC estimators based on Bernstein polynomials have become increasingly popular (see [15,16]). This estimator provides the advantages of achieving asymptotic convergence and producing a smooth ROC curve estimate. Among its drawbacks are poor boundary accuracy, limited flexibility, and the need for high-degree polynomials for analyzing complex data sets. The literature uses general versions of Bernstein polynomials to overcome these limitations.

Many ways exist to generalize Bernstein polynomials [17–19], with the Non-Uniform Rational B-spline (NURBS) being one of the most striking. A NURBS curve devoid of interior knots simplifies to the Bernstein polynomial when the weight values are equal. NURBS facilitates the precise design of curves and surfaces by including interior knots and weights into the model; hence, it enables more accurate control over the geometry of the planned object. In this study, we introduce a novel non-parametric estimation method for the ROC curve using NURBS. NURBS considers weights, control points, and knot sequences in the statistical model formulation. We obtain the weights by minimizing the difference between the suggested estimator and the empirical ROC estimator. We compute the number of interior knots using the Akaike Information Criterion (AIC), a common tool in statistical model selection. We determine control points using the empirical ROC estimator. Then, we demonstrate that the new NURBS estimator can be simplified to the Bernstein polynomials method proposed by [15] if we select a knot sequence where both 0 and 1 occur m+1 times when the weight values are the same. Finally, we design simulation studies to test the performance of the NURBS method. We compare it with Bernstein polynomials and the empirical ROC estimator in terms of averaged squared errors (ASE).

The remainder of this paper is organized as follows: First, we introduce the framework of the proposed NURBS-based estimate approach for the ROC curve. We also analyze the determination of *w*_*k*_ weights and a strategy for selecting the number of interior knots using the AIC measure. In the next section, we conduct an extensive simulation analysis that evaluates our estimator against Bernstein polynomials, the empirical ROC estimator, and the kernel-based ROC estimator. We also evaluate the efficacy of our estimator using actual medical datasets. Finally, we present the conclusion.

## Estimation of the ROC curve using non-uniform rational B-spline

Bernstein polynomials provide a strong method for estimating the ROC curve because they are among the simplest polynomial approximations incorporating a probabilistic interpretation. [15] presented a non-parametric estimating approach utilizing Bernstein polynomials for the estimation of the ROC curve. This approach relies solely on control points, so it has specific inadequacies for shape control. This paper presents a new non-parametric approach for estimating the ROC curve utilizing NURBS. The proposed NURBS methodology in this study extends the approach designed in [15]. While [15]’s model relies solely on control points, the NURBS method improves estimation accuracy by adding a knot sequence and weights to the model. NURBS allows for better shape flexibility by using control points, weights, and the knot sequence together to create more accurate estimations on complex data sets. Using the mathematical benefits of the knot sequence and rational polynomials in NURBS allows for more flexible and versatile estimates.

We aim to provide a smooth estimate of the ROC curve, which is defined as $ROC(t)=1\;-\;G(F^{-1}(1\;-\;t))$. We present the NURBS estimator of degree *m* > 0 for the ROC curve as a generalization of the classical Bernstein estimator by

$${\hat{ROC}}_{m,w,t}(t)=\sum_{k=0}^{m+l-1}ROC_{n}\left(\theta_{k}\right)R_{k,m}(t)$$ (3)

where


$$R_{k,m}(t)=\frac{w_{k}N_{k,m}(t)}{\sum_{j=0}^{m+l-1}w_{j}N_{j,m}(t)}$$

are the rational B-spline basis functions. The control points are defined by $ROC_{n}(\theta_{k})$, where $\theta_{k}$ are the averaged knot positions calculated as:


$$\theta_{k}=\frac{1}{m}\sum_{i=1}^{m}t_{i+k},\;k=0,1,\ldots ,m+l-1,$$

and the total number of control points is m+l, where *l*–1 is the number of interior knots. This estimator is designed for continuous functions between 0 and 1 and depends on two positive whole numbers, *l* and *m*, along with a knot sequence that is non-decreasing with


$$T_{l,m}=\left(\underset{m+1\text{ times}}{\underbrace{t_{0}=\cdots =t_{m}}}=0<t_{m+1}<\cdots <t_{m+l-1}<\underset{m+1\text{ times}}{\underbrace{t_{m+l}=\cdots =t_{2m+l}}}=1\right).$$

The knot sequence defines the B-spline basis functions of degree *m*, denoted as *N*_*k*,*m*_(*t*), which are expressed recursively as follows:


$$N_{k,0}(t)=\left\{ \begin{array}{cc} 1, & \text{if }t\in [t_{k},t_{k+1})\\ 0, & \text{otherwise} \end{array}\right.$$

$$N_{k,m}(t)=\frac{t-t_{k}}{t_{k+m}-t_{k}}N_{k,m-1}(t)+\frac{t_{k+m+1}-t}{t_{k+m+1}-t_{k+1}}N_{k+1,m-1}(t)$$ (4)

for k=0,…,m+l−1 and m≥1. Furthermore, we can derive the following equations for the proposed ROC estimator for m≥1:

$${\hat{ROC}}_{m,w,t}(0)=ROC(0)=0\;and\;{\hat{ROC}}_{m,w,t}(1)=ROC(1)=1.$$ (5)

Additionally, to ensure monotonic and non-decreasing behaviors consistent with the ROC curve, the following conditions must be satisfied: $w_{k}\;>\;0$, for k=0,1,…,m+l−1, and $ROC_{n}(\theta_{k+1})-ROC_{n}(\theta_{k})\geq 0$, where k=0,1,…,m+l−2 (for further details, see [20]).

Under specific conditions, the proposed estimator reduces to the ROC estimator based on Bernstein polynomials, as described by [15]. This reduction occurs when all weights are equal and no interior knots are used (which means that *l* = 1). In this case, the knot sequence simplifies to


$$T_{l,m}=\left(0,\ldots ,0,1,\ldots ,1\right),$$

where 0 and 1 each appear *m* + 1 times.

### Determination of knot sequence

The knot sequence is crucial to the efficacy of the suggested estimate approach. The determination of the knot sequence reveals two particular issues: the selection of the number of interior knots and their positioning. The study by [21] establishes a foundation for addressing these issues. We select a knot sequence that evenly distributes interior knots across the percentile rankings, in accordance with the research of [21]. Furthermore, we determine the ideal number of interior knots by using the AIC criterion detailed below.

$$(l-1{)}^{\text{opt}}=\arg\min_{\left(l-1\right)}\left\{\log\left((1/N)\sum_{i=1}^{N}{\left(ROC_{n}(t_{i})-{\hat{ROC}}_{m,w,t}(t_{i})\right)}^{2}\right)+\frac{2(m+l)}{N}\right\}$$ (6)

where m+l denotes the number of control points and *N* represents the number of evaluation points on the empirical ROC curve.

### The obtaining of the weights *w*_*k*_

A weighted sum of NURBS basis functions can approximate the unknown ROC curve. The control points, weights (*w*_*k*_), and knot sequence utilized in the NURBS model facilitate flexible fitting of the ROC curve. We estimate the *w*_*k*_ coefficients by solving the following optimization equation, which is derived from the empirical ROC estimator.

$$\hat{w}={arg\;min}_{w}\sum_{i=1}^{N}{\left[ROC_{n}(t_{i})-{\hat{ROC}}_{m,w,t}(t_{i})\right]}^{2}$$ (7)

We outline the ROC estimator using NURBS as follows:

$${\hat{ROC}}_{m,w,t}(t)=\sum_{k=0}^{m+l-1}ROC_{n}\left(\theta_{k}\right)\frac{\hat{w_{k}}N_{k,m}(t)}{\sum_{j=0}^{m+l-1}\hat{w_{j}}N_{j,m}(t)}$$ (8)

The following conditions must hold for the function to be continuous and monotonic:

1.
$w_{k}>0,\;k=0,1,\ldots ,m+l-1.$

2. $ROC_{n}(\theta_{k+1})-ROC_{n}(\theta_{k})\geq 0$, k=0,1,...,m+l−2.

To solve the constrained optimization problem, we employ the “L-BFGS-B” algorithm implemented in R’s optim function, which supports box-constrained quasi-Newton optimization. Each weight is bounded below by 10^−6^ to ensure numerical stability and strictly positive rational basis functions. The optimization minimizes the sum of squared differences between the empirical ROC curve and the estimated NURBS-based ROC curve at observed FPR points. The B-spline basis functions *N*_*k*,*m*_(*t*) are computed using the splines package, where interior knots are placed at equally spaced percentile rankings of the false positive rates. To avoid overfitting, the number of interior knots is selected by minimizing the Akaike Information Criterion (AIC).

## Simulation studies

This section examines the performance of the proposed NURBS-based ROC estimator in comparison with three benchmark estimators: the empirical ROC curve, the Bernstein polynomial-based estimator, and the kernel-based ROC estimator proposed by [22], implemented using the kroc() function from the R ks package. We conduct comprehensive Monte Carlo simulations on different sample sizes, *n*_1_ and *n*_2_, for comparative analysis. The simulation studies consist of six scenarios, each consisting of different distribution combinations for the random variables *X* and *Y*, as illustrated below:

1. *S*_1_: X~Normal(0,1),Y~Normal(1,1)
2. *S*_2_: X~Normal(0,1),Y~Normal(2,1.2)
3. *S*_3_: X~Exponential(2),Y~Exponential(1)
4. *S*_4_: X~Gamma(0.5,1),Y~Gamma(0.5,4)
5. *S*_5_: X~Normal(2,1),Y~Gamma(2,2)
6. *S*_6_: X~Normal(1,1),Y~Gamma(2,2)

The *S*_5_ and *S*_6_ scenarios are significant because, in practice, the distribution often shifts from symmetric to right-skewed when individuals are sick. For each ROC curve, including equal or unequal sample sizes, we carry out 1000 Monte Carlo simulations: $\left(n_{1},n_{2})\in (25,15),(20,20),(15,25),(60,40),(50,50),(40,60),(125,75),(100,100),(75,125\right)$. We then investigate how well the empirical ROC estimator *ROC*_*n*_, the Bernstein ROC estimator *ROC*_*m*_(*t*), the kernel-based ROC estimator kROC, and the NURBS ROC estimator ${\hat{ROC}}_{m,w,t}(t)$ perform for each dataset. To assess model complexity in ROC curve estimation, we select polynomial degrees m=2,4,6 for Bernstein and NURBS estimators. These degrees span low to moderate complexity, enabling systematic evaluation of model flexibility while balancing computational efficiency. Lower degrees are too restrictive for ROC curve patterns, while higher degrees risk overfitting in small samples. Fixed degrees ensure consistent method comparisons. We use eight different ways to estimate the ROC curve: the empirical ROC estimator *ROC*_*n*_(*t*), the kernel-based ROC estimator (kROC), the Bernstein polynomial of degree 2 (BP2), the Bernstein polynomial of degree 4 (BP4), the Bernstein polynomial of degree 6 (BP6), the NURBS of degree 2 (NB2), the NURBS of degree 4 (NB4), and the NURBS of degree 6 (NB6). We evaluate the accuracy of the eight estimators using the averaged squared errors (ASE). We calculate ASE to evaluate the quality of the estimators as follows:

$$ASE=(1/N)\sum_{i=1}^{N}(\hat{ROC}(t_{i})-ROC(t_{i}){)}^{2}$$ (9)

where $\hat{ROC}(t)$ denotes the estimated ROC curves. A lower ASE value for an estimator indicates a better approximation of the true ROC curve.

Tables 1 and 2 present the ASE values for eight different ROC curve estimation methods across six simulation scenarios involving diverse distributional structures and varying sample sizes. These results provide a comprehensive assessment of estimator performance under both symmetric and asymmetric or heavy-tailed settings. Across various sample size settings and particularly under skewed or heterogeneous distributional scenarios, the proposed NURBS estimators (especially NB4 and NB6) frequently yield the lowest ASE values, indicating superior approximation accuracy. However, in certain scenarios involving small sample sizes, kernel-based or even Bernstein estimators may deliver comparable or superior performance. The kROC estimator performs competitively in symmetric distributions, often surpassing the empirical and BP estimators. However, its performance declines in more complex distributional settings, suggesting limitations in handling complexity or skewness. BP estimators exhibit a consistent trend of improvement with increasing polynomial degree. Nonetheless, even the highest-degree BP estimator (BP6) typically falls short of matching the accuracy of the NURBS estimators of the same degree, underscoring the additional flexibility introduced by weights and interior knots in the NURBS framework. The empirical ROC estimator ${ROC}_{n}(t)$ consistently underperforms across all scenarios, reflecting its lack of smoothing and structure.

**Table 1:** ASE values of eight different estimators for the first three different scenarios.

Distributions	($n_{1}$, $n_{2}$)	BP2	NB2	BP4	NB4	BP6	NB6	$ROC_{n}(t)$	kROC
X~Normal(0,1)	(25,15)	0.0320	0.0127	0.0136	0.0126	0.0096	0.0125	0.0187	0.0097
Y~Normal(1,1)	(20,20)	0.0308	0.0113	0.0120	0.0112	0.0096	0.0113	0.0196	0.0089
	(15,25)	0.0320	0.0116	0.0151	0.0116	0.0116	0.0117	0.0249	0.0094
	(60,40)	0.0307	0.0057	0.0111	0.0057	0.0069	0.0057	0.0072	0.0044
	(50,50)	0.0312	0.0059	0.0119	0.0059	0.0072	0.0058	0.0078	0.0046
	(40,60)	0.0315	0.0061	0.0116	0.0061	0.0078	0.0061	0.0090	0.0049
	(125,75)	0.0312	0.0031	0.0111	0.0031	0.0064	0.0031	0.0036	0.0025
	(100,100)	0.0316	0.0031	0.0113	0.0031	0.0066	0.0031	0.0037	0.0025
	(75,125)	0.0326	0.0035	0.0124	0.0035	0.0070	0.0035	0.0045	0.0028
X~Normal(0,1)	(25,15)	0.0966	0.0063	0.0477	0.0063	0.0301	0.0063	0.0359	0.0119
Y~Normal(2,1.2)	(20,20)	0.0928	0.0051	0.0459	0.0051	0.0297	0.0052	0.0463	0.0094
	(15,25)	0.0868	0.0049	0.0443	0.0050	0.0278	0.0050	0.0639	0.0077
	(60,40)	0.1066	0.0036	0.0558	0.0035	0.0370	0.0035	0.0181	0.0061
	(50,50)	0.1076	0.0037	0.0582	0.0036	0.0391	0.0036	0.0237	0.0056
	(40,60)	0.1074	0.0037	0.0586	0.0036	0.0403	0.0036	0.0309	0.0052
	(125,75)	0.1104	0.0022	0.0589	0.0023	0.0401	0.0023	0.0093	0.0041
	(100,100)	0.1136	0.0024	0.0632	0.0024	0.0440	0.0024	0.0132	0.0038
	(75,125)	0.1155	0.0026	0.0668	0.0026	0.0467	0.0026	0.0196	0.0038
X~Exponential(2)	(25,15)	0.0175	0.0136	0.0108	0.0135	0.0097	0.0135	0.0177	0.0126
Y~Exponential(1)	(20,20)	0.0163	0.0121	0.0090	0.0119	0.0086	0.0120	0.0173	0.0110
	(15,25)	0.0178	0.0116	0.0109	0.0115	0.0098	0.0116	0.0192	0.0107
	(60,40)	0.0148	0.0062	0.0069	0.0061	0.0054	0.0061	0.0072	0.0061
	(50,50)	0.0149	0.0056	0.0072	0.0056	0.0053	0.0056	0.0070	0.0058
	(40,60)	0.0147	0.0055	0.0066	0.0055	0.0053	0.0055	0.0075	0.0058
	(125,75)	0.0150	0.0031	0.0067	0.0031	0.0046	0.0031	0.0035	0.0039
	(100,100)	0.0149	0.0028	0.0065	0.0029	0.0044	0.0028	0.0033	0.0037
	(75,125)	0.0153	0.0029	0.0069	0.0029	0.0046	0.0029	0.0037	0.0039

**Table 2:** ASE values of eight different estimators for the last three different scenarios.

Distributions	($n_{1}$, $n_{2}$)	BP2	NB2	BP4	NB4	BP6	NB6	$ROC_{n}(t)$	kROC
X~Gamma(0.5,1)	(25,15)	0.0277	0.0131	0.0158	0.0129	0.0126	0.0129	0.0202	0.0214
Y~Gamma(0.5,4)	(20,20)	0.0255	0.0105	0.0134	0.0104	0.0111	0.0104	0.0198	0.0193
	(15,25)	0.0260	0.0097	0.0143	0.0097	0.0110	0.0097	0.0233	0.0181
	(60,40)	0.0263	0.0049	0.0128	0.0048	0.0090	0.0048	0.0075	0.0128
	(50,50)	0.0263	0.0045	0.0133	0.0045	0.0091	0.0045	0.0080	0.0128
	(40,60)	0.0262	0.0042	0.0128	0.0042	0.0092	0.0042	0.0089	0.0129
	(125,75)	0.0270	0.0028	0.0133	0.0028	0.0090	0.0028	0.0039	0.0091
	(100,100)	0.0271	0.0024	0.0134	0.0024	0.0092	0.0024	0.0040	0.0093
	(75,125)	0.0275	0.0023	0.0140	0.0024	0.0094	0.0023	0.0048	0.0098
X~Normal(2,1)	(25,15)	0.0371	0.0113	0.0215	0.0109	0.0160	0.0109	0.0273	0.0183
Y~Gamma(2,2)	(20,20)	0.0334	0.0081	0.0182	0.0078	0.0138	0.0078	0.0285	0.0144
	(15,25)	0.0314	0.0072	0.0173	0.0069	0.0123	0.0070	0.0334	0.0121
	(60,40)	0.0386	0.0043	0.0218	0.0043	0.0156	0.0042	0.0133	0.0143
	(50,50)	0.0375	0.0034	0.0215	0.0034	0.0152	0.0034	0.0148	0.0125
	(40,60)	0.0360	0.0030	0.0203	0.0031	0.0146	0.0030	0.0176	0.0110
	(125,75)	0.0404	0.0023	0.0230	0.0023	0.0163	0.0023	0.0083	0.0126
	(100,100)	0.0397	0.0018	0.0230	0.0018	0.0165	0.0019	0.0101	0.0113
	(75,125)	0.0388	0.0016	0.0229	0.0016	0.0163	0.0016	0.0127	0.0104
X~Normal(1,1)	(25,15)	0.0746	0.0061	0.0369	0.0058	0.0239	0.0058	0.0394	0.0215
Y~Gamma(2,2)	(20,20)	0.0689	0.0046	0.0333	0.0043	0.0220	0.0043	0.0489	0.0168
	(15,25)	0.0634	0.0039	0.0313	0.0037	0.0197	0.0038	0.0624	0.0136
	(60,40)	0.0825	0.0027	0.0434	0.0027	0.0298	0.0026	0.0245	0.0165
	(50,50)	0.0807	0.0023	0.0437	0.0023	0.0298	0.0022	0.0303	0.0145
	(40,60)	0.0779	0.0022	0.0420	0.0021	0.0292	0.0020	0.0377	0.0129
	(125,75)	0.0872	0.0015	0.0473	0.0015	0.0332	0.0015	0.0165	0.0151
	(100,100)	0.0865	0.0013	0.0484	0.0013	0.0346	0.0013	0.0223	0.0136
	(75,125)	0.0849	0.0011	0.0488	0.0012	0.0348	0.0012	0.0298	0.0123

For a statistically rigorous comparison, the performance of NB6 against BP6 and kROC for the sample size $\left(n_{1},n_{2})=(100,100\right)$ is analyzed, as reported in Tables 3 and 4. NB6 and BP6 are selected because they generally yield lower ASE values compared to their lower-degree counterparts (NB2, NB4, BP2, BP4), particularly in larger sample sizes and complex distributional scenarios. The sample size $\left(n_{1},n_{2})=(100,100\right)$ is chosen because it provides a balanced, moderately large dataset that ensures sufficient statistical power for reliable estimation while maintaining computational efficiency, a common scenario in diagnostic testing applications. The inclusion of 95% bootstrap confidence intervals (CIs) and paired t-test p-values in Tables 3 and 4 provides an assessment of performance differences. All 95% confidence intervals are computed using nonparametric bootstrap with *B* = 1000 resampling iterations. Comprehensive ASE values with 95% bootstrap CIs for all estimators, scenarios, and sample sizes are provided in the supplementary S1 Table, enabling a thorough evaluation of estimator accuracy and variability.

**Table 3:** Comparison of ASE values with 95% bootstrap confidence intervals (CIs) and paired *t*-test *p*-values for NB6 vs BP6 estimators under equal sample sizes ($n_{1}=n_{2}=100$).

Scenario	($n_{1}$, $n_{2}$)	BP6 (95% CI)	NB6 (95% CI)	*p*-value
*S*_1_	(100, 100)	0.0066 (0.0063, 0.0068)	0.0031 (0.0029, 0.0033)	*p* < 0.001
*S*_2_	(100, 100)	0.0440 (0.0434, 0.0445)	0.0024 (0.0022, 0.0025)	*p* < 0.001
*S*_3_	(100, 100)	0.0044 (0.0042, 0.0046)	0.0028 (0.0027, 0.0030)	*p* < 0.001
*S*_4_	(100, 100)	0.0092 (0.0090, 0.0093)	0.0024 (0.0023, 0.0025)	*p* < 0.001
*S*_5_	(100, 100)	0.0165 (0.0163, 0.0168)	0.0019 (0.0018, 0.0020)	*p* < 0.001
*S*_6_	(100, 100)	0.0346 (0.0341, 0.0350)	0.0013 (0.0012, 0.0014)	*p* < 0.001

**Table 4:** Comparison of ASE values with 95% bootstrap confidence intervals (CIs) and paired *t*-test *p*-values for NB6 vs kROC estimators under equal sample sizes ($n_{1}=n_{2}=100$).

Scenario	($n_{1}$, $n_{2}$)	kROC (95% CI)	NB6 (95% CI)	*p*-value
*S*_1_	(100, 100)	0.0025 (0.0024, 0.0027)	0.0031 (0.0029, 0.0033)	*p* < 0.001
*S*_2_	(100, 100)	0.0038 (0.0036, 0.0041)	0.0024 (0.0022, 0.0025)	*p* < 0.001
*S*_3_	(100, 100)	0.0037 (0.0035, 0.0039)	0.0028 (0.0027, 0.0030)	*p* < 0.001
*S*_4_	(100, 100)	0.0093 (0.0089, 0.0096)	0.0024 (0.0023, 0.0025)	*p* < 0.001
*S*_5_	(100, 100)	0.0113 (0.0110, 0.0117)	0.0019 (0.0018, 0.0020)	*p* < 0.001
*S*_6_	(100, 100)	0.0136 (0.0132, 0.0140)	0.0013 (0.0012, 0.0014)	*p* < 0.001

Table 3 shows that NB6 consistently achieves lower ASE values than BP6 across all six scenarios, with non-overlapping 95% CIs indicating clear performance differences. The paired t-test p-values (*p* < 0.001) confirm the statistical significance of these differences across all balanced sample sizes evaluated ($n_{1}=n_{2}=100$). This improved performance results from the NURBS framework’s incorporation of weights and an interior knot vector at fixed polynomial degree, which increases flexibility and allows better adaptation to complex distributional shapes when sufficient data are available.

Table 4 compares NB6 with kROC. In scenario *S*_1_, kROC exhibits a lower ASE value than NB6, with a statistically significant difference (*p* < 0.001), indicating better performance in this symmetric distribution. In the remaining scenarios (*S*_2_–*S*_6_), NB6 consistently achieves significantly lower ASE values (*p* < 0.001), demonstrating improved performance across diverse distributional structures.

In addition to fixed polynomial degrees (m=2,4,6), we also consider an adaptive selection of degree *m* based on sample size, motivated by the theoretical recommendation in [15]. Specifically, we follow the guideline $m=0.5\;\cdot \;n_{2}^{2/3}$, as suggested by their analysis, to determine the optimal degree in a data-driven manner. This approach balances approximation accuracy with model complexity and ensures adaptivity to varying sample sizes. The resulting estimators are referred to as BP($m_{opt}$) and NB($m_{opt}$) throughout the study.

To further assess the effect of data-driven model complexity, we implemented the adaptive degree selection rule $m=0.5\;\cdot \;n_{2}^{2/3}$ proposed by [15], resulting in estimators denoted as BP($m_{opt}$) and NB($m_{opt}$). ASE values and 95% bootstrap confidence intervals for these estimators are computed under all scenarios and sample sizes. While full results are reported in Supplementary S1 Table, a subset of these findings is summarized in Table 5 for the equal-sample-size case ($n_{1}=n_{2}=100$).

**Table 5:** Comparison of ASE values with 95% bootstrap confidence intervals (CIs) and paired *t*-test *p*-values for BP($m_{opt}$) and NB($m_{opt}$) under equal sample sizes ($n_{1}=n_{2}=100$).

Scenario	($n_{1}$, $n_{2}$)	BP($m_{opt}$) (95% CI)	NB($m_{opt}$) (95% CI)	*p*-value
*S*_1_	(100, 100)	0.0031 (0.0029, 0.0033)	0.0031 (0.0029, 0.0033)	*p* = 0.747
*S*_2_	(100, 100)	0.0238 (0.0233, 0.0242)	0.0024 (0.0022, 0.0025)	*p* < 0.001
*S*_3_	(100, 100)	0.0028 (0.0026, 0.0030)	0.0028 (0.0027, 0.0030)	*p* = 0.576
*S*_4_	(100, 100)	0.0052 (0.0050, 0.0053)	0.0024 (0.0023, 0.0025)	*p* < 0.001
*S*_5_	(100, 100)	0.0098 (0.0096, 0.0100)	0.0018 (0.0017, 0.0019)	*p* < 0.001
*S*_6_	(100, 100)	0.0202 (0.0198, 0.0205)	0.0013 (0.0012, 0.0013)	*p* < 0.001

As shown in Table 5, NB($m_{opt}$) generally yields lower ASE values than BP($m_{opt}$) across all six scenarios. The improvement is especially notable in *S*_4_, *S*_5_, and *S*_6_, which involve asymmetric or heavy-tailed distributions, and also in *S*_2_, where both distributions are symmetric but have different variances. In these cases, the differences are statistically significant (*p* < 0.001). In the remaining scenarios (*S*_1_ and *S*_3_), NB($m_{opt}$) performs similarly to BP($m_{opt}$), with minor differences and no statistical significance. These results show that NB($m_{opt}$) adapts well to complex distributions and performs at least as reliably as the classical Bernstein estimator.

The comprehensive results in S1 Table, which include ASE values and 95% bootstrap CIs for all estimators across all scenarios and sample sizes, confirm that NURBS estimators—particularly NB4, NB6, and the data-adaptive NB($m_{opt}$)—consistently outperform classical methods, especially in larger samples and under complex distributional settings. These findings demonstrate the effectiveness of the NURBS-based ROC estimator as a flexible and accurate nonparametric alternative. Its ability to maintain strong performance even at small or moderate degrees makes it well-suited for practical applications.

### Real data examples

### Metastatic kidney cancer data

This section assesses the effectiveness of our proposed method utilizing an actual data set from [15]. A clinical investigation conducted from November 2008 to August 2011 resulted in the collection of this dataset, collected by a research team led by Dr. Krzysztof Tupikowski from the Department of Urology and Oncological Urology at the Medical University of Wroclaw [6]. The primary objective of their study was to evaluate the presence of predictive indicators for the response to their newly proposed treatment method. This dataset includes two biomarkers: serum fibrinogen concentration (FC) and hemoglobin level (HL). Data were collected from 31 patients $n_{1}=17,n_{2}=14$ for HL; $n_{1}=12,n_{2}=14$ for FC. The study assessed each patient’s clinical response as either presence (1) or absence (0) at week 24.

**Fig 1:**
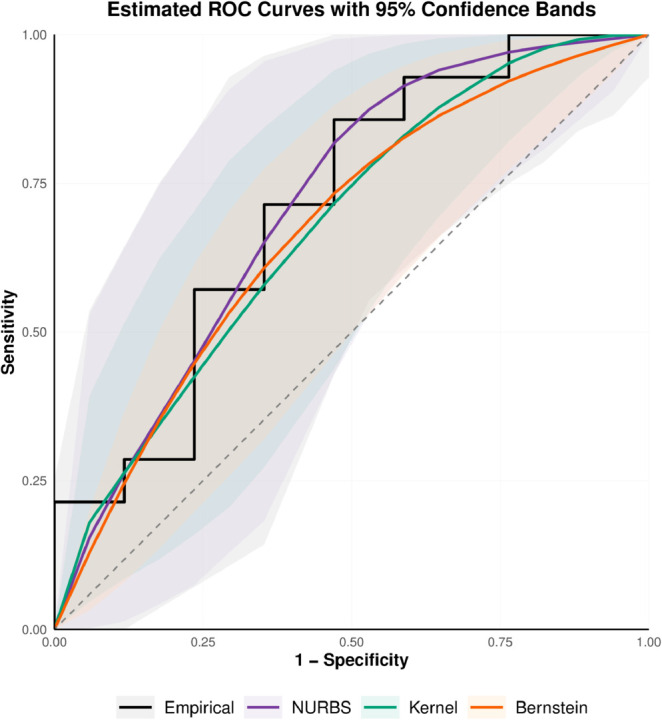
Estimated ROC curves with 95% bootstrap confidence bands for four nonparametric methods based on the HL dataset.

**Fig 2:**
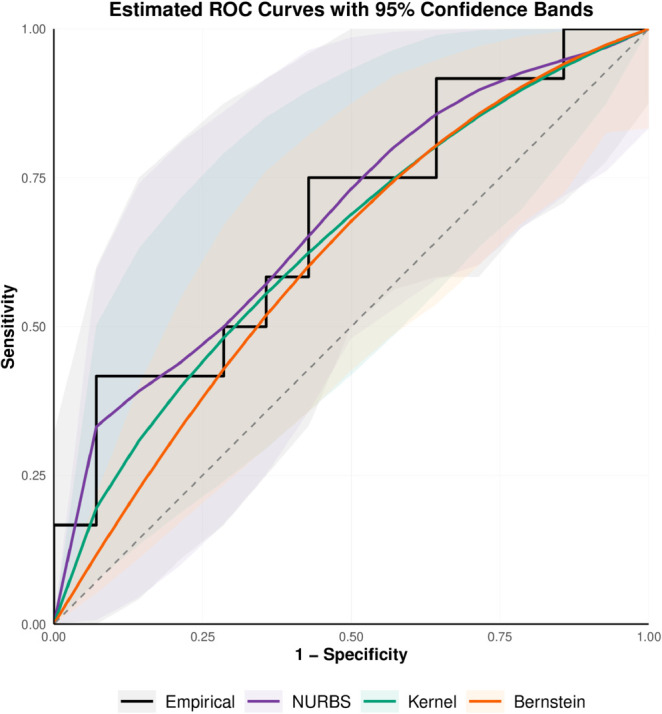
Estimated ROC curves with 95% bootstrap confidence bands for four nonparametric methods based on the FC dataset.

We employ ROC curve estimation techniques on this data to determine if HL and FC indicate treatment response in patients with metastatic kidney cancer. We analyze the HL and FC data to investigate how well the NURBS-based ROC estimator performs against the empirical ROC, the Bernstein polynomial estimators, and the kernel-based estimator. Because FC serves as a negative predictor of treatment response, the ROC analysis accounts for this inverse relationship between FC levels and treatment outcomes. In this study, we estimate the true ROC curve using the empirical ROC estimator, the classical Bernstein estimator of degree four, the NURBS estimator of degree four, and the kernel-based ROC (kROC) estimator. The ROC curve estimations along with their 95% bootstrap confidence intervals are presented in Figs 1 and 2. We obtain HL’s AUC values of 0.718, 0.670, 0.711, and 0.675 and FC’s values of 0.690, 0.616, 0.680, and 0.640, respectively, utilizing the empirical ROC curve estimator, the classical Bernstein estimator, the NURBS estimator, and the kernel-based estimator.

The estimator that uses the Bernstein polynomial gives a lower AUC value, and the empirical ROC estimator is non-smooth when the sample sizes are small, as shown in Figs 1 and 2. The results suggest that the Bernstein ROC estimator requires more degrees since a four-degree polynomial is insufficient. The NURBS-based ROC estimator has a smooth shape and works like the empirical ROC estimator by adding the weights *w*_*k*_ and interior knots in the model. The kernel-based ROC estimator also produces a smooth curve but tends to slightly underestimate the AUC compared to the empirical estimator.

### Diffuse large B-Cell lymphoma data

In this study, the Diffuse Large B-Cell Lymphoma (DLBCL) dataset, created by [23] and frequently used in biomarker selection and classification studies, has been analyzed. DLBCL and follicular lymphoma (FL) are B-cell malignancies that differ significantly in their clinical features, natural histories, and responses to therapy. The goal of the dataset is to distinguish between these two types of lymphoma using gene expression data. The dataset consists of 58 DLBCL and 19 FL samples, with gene expression levels measured for 7070 genes in each sample.

In our analysis, we focus on two genes—U46006_s_at and U96113_at —which have been highlighted in previous studies as prominent markers in gene expression analyses related to diffuse large B-cell lymphoma (DLBCL). These genes are among the commonly used biomarkers for the diagnosis of hematological malignancies, as emphasized in prior research, particularly by [24].

To assess the effectiveness of different ROC curve estimation techniques in the context of biomarker evaluation, we apply four methods: the empirical ROC estimator, the classical Bernstein polynomial estimator of degree two, the NURBS-based ROC estimator of degree two, and the kernel-based ROC estimator. These methods are employed to model the diagnostic accuracy of the selected genes, U46006_s_at and U96113_at, in differentiating between DLBCL and FL.

**Fig 3:**
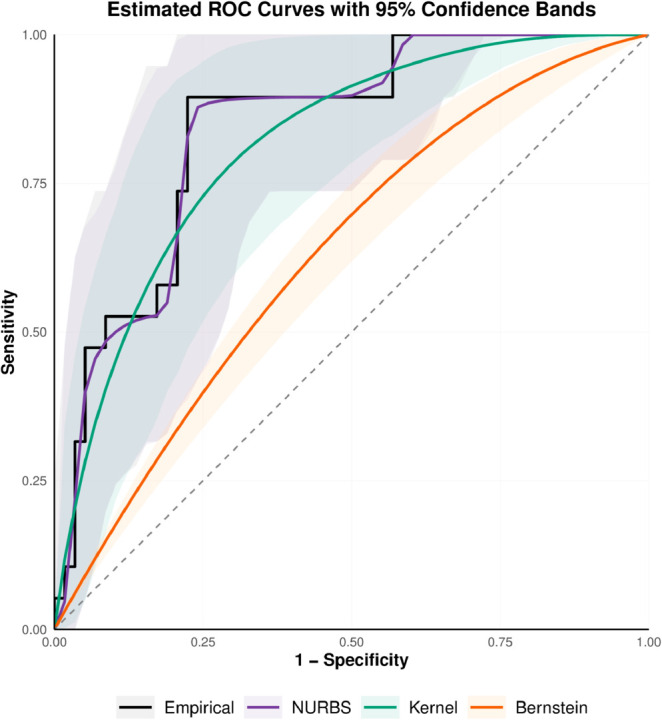
Estimated ROC curves with 95% bootstrap confidence bands for four nonparametric methods applied to the U46006_s_at gene.

**Fig 4:**
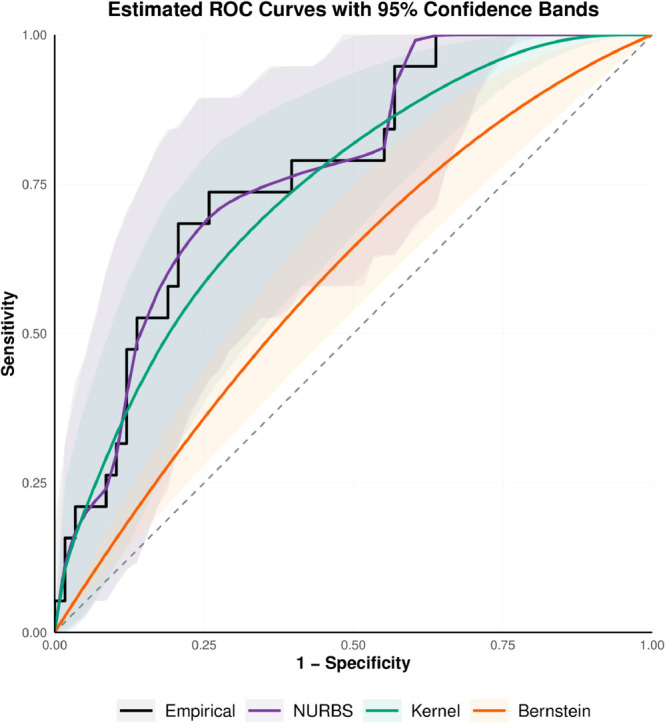
Estimated ROC curves with 95% bootstrap confidence bands for four nonparametric methods applied to the U96113_at gene.

The comparative results of the estimators along with their 95% bootstrap confidence intervals are depicted in Figs 3 and 4. For the U46006_s_at gene, the AUC values from the empirical, classical Bernstein, the NURBS, and the kernel-based estimators are 0.843, 0.632, 0.839, and 0.814, respectively. For the U96113_at gene, the corresponding AUC values obtained using the same estimators are 0.772, 0.596, 0.773, and 0.738.

As expected, the empirical ROC estimator, while non-parametric and simple, produces a non-smooth curve, particularly with moderate sample sizes. The classical Bernstein estimator, though smoother, tends to underestimate the true AUC due to its limited flexibility at lower degrees. The kernel-based estimator generates a smooth curve but slightly underestimates the AUC compared to the empirical estimator. In contrast, the NURBS-based estimator provides a smooth and adaptive approximation of the ROC curve by introducing additional the weights *w*_*k*_ and interior knots, which enhance the model to closely follow the empirical distribution while preserving smoothness.

Overall, our findings indicate that the NURBS-based approach achieves AUC values that are comparable to or better than those of the empirical, kernel-based, and Bernstein estimators, highlighting its potential as an effective alternative for ROC curve estimation in high-dimensional gene expression analyses.

## Practical application guide

To facilitate the practical application of the proposed NURBS-based ROC curve estimation method, we provide a step-by-step guide for practitioners.

### Data requirements

The proposed estimator requires two numeric samples representing:

- *X*: test measurements for the healthy (non-diseased) group
- *Y*: test measurements for the diseased group

Both *X* and *Y* should contain continuous values.

### Software environment

The proposed algorithm can be implemented in any scientific computing environment that supports B-spline basis computation and constrained nonlinear optimization. In this study, we provide an implementation in the R programming language as an example. The R version utilizes the following standard packages:

- splines (for B-spline basis computation)
- pROC (for empirical ROC estimation)
- optim (for weight optimization; part of base R)

### Estimation procedure

The estimation process consists of the following steps:

1. **Compute the empirical ROC curve:** The empirical ROC curve is calculated using standard ROC estimation software such as the pROC package.
2. **Determine interior knots:** Interior knots are selected based on equally spaced percentiles of the empirical false positive rates.
3. **Calculate control point locations:** Control point locations are obtained from knot averages, and their corresponding values are calculated from the empirical ROC evaluated at these locations.
4. **Estimate optimal weights:** The weights *w*_*k*_ are obtained by minimizing the squared difference between the empirical ROC curve and the NURBS-based estimator, subject to positivity constraints. This optimization is performed using the L-BFGS-B algorithm implemented in the optim function.
5. **Select the number of interior knots:** The optimal number of interior knots is selected using the Akaike Information Criterion (AIC) to balance goodness-of-fit and model complexity.
6. **Compute the NURBS-based ROC curve:** The final ROC curve is constructed using the estimated knots, control points, and weights.

## Conclusion

This study presents a flexible method for ROC curve estimation using the NURBS approach, which extends the Bernstein polynomial technique proposed by [15]. While Bernstein-based estimators provide smooth approximations, they are limited by fixed knot structures and lack of local control. The proposed NURBS method addresses these limitations by incorporating flexible interior knots and weights, allowing for greater adaptability and precision in curve modeling.

The performance of the proposed estimator is comprehensively evaluated through Monte Carlo simulation studies and analyses of two real-world medical datasets. Across most scenarios, the NURBS-based estimator generally yields lower ASE values compared to the Bernstein, empirical, and kernel-based ROC estimators, particularly under complex and asymmetric distributional structures. The flexibility introduced by interior knots and adaptive weights enables the NURBS framework to more accurately approximate the true ROC curve while maintaining smoothness. Applications to metastatic kidney cancer and diffuse large B-cell lymphoma datasets further demonstrate the practical utility of the NURBS estimator, providing smooth and stable estimates that closely align with the empirical ROC curve. Notably, strong performance is observed even at relatively low polynomial degrees due to the method’s local adaptability and structural flexibility.

Although this study primarily focuses on biostatistical applications, the NURBS-based ROC estimator may also be applicable to other fields involving binary classification or predictive modeling. Examples include machine learning, psychology, social sciences, econometrics, financial risk assessment, bioinformatics, environmental risk modeling, and cybersecurity, where accurate evaluation of classification performance is essential. The methodological framework provided here may serve as a reference for researchers working in these domains.

Nevertheless, certain limitations should be noted. The NURBS-based method may be computationally demanding for very large datasets due to the optimization involved in weight estimation. Moreover, selecting the number of interior knots remains a crucial step, as improper selection may lead to potential overfitting, especially in small sample scenarios.

Overall, the proposed estimator offers a statistically strong and flexible alternative for ROC curve estimation. Its consistent performance across different scenarios highlights its potential as a valuable tool in diagnostic accuracy analysis.

## Supporting information

S1 Table ASE values and 95% bootstrap confidence intervals for all estimators under all scenarios. The complete table is provided as a supplementary file due to its large size.
(XLSX)

S1 File R source code for real data applications using the proposed NURBS-based ROC estimator. This file contains the full R implementation of the NURBS-based ROC curve estimation method applied to the real datasets analyzed in this study.
(R)
